# Association of the Spectrum of Cutaneous Lupus Erythematosus with Disease Activity and Systemic Manifestations in Patients with Systemic Lupus Erythematosus

**DOI:** 10.31138/mjr.200423.aos

**Published:** 2023-09-19

**Authors:** Herwinda Brahmanti, Cesarius Singgih Wahono, Mirza Zaka Pratama, Perdana Aditya Rahman, Riski Bagus Suhendra, Amalia Novia Rizky, Nabilah Hanifah Mukti

**Affiliations:** 1 Department of Dermatology and Venereology, Faculty of Medicine, Universitas Brawijaya – RSUD dr. Saiful Anwar Malang, Indonesia,; 2 Rheumatology Division, Department of Internal Medicine, Faculty of Medicine, Universitas Brawijaya – RSUD dr. Saiful Anwar Malang, Indonesia

**Keywords:** cutaneous manifestation, systemic lupus erythematosus, disease activity, cutaneous lupus

## Abstract

**Background:**

Cutaneous involvement is common in systemic lupus erythematosus (SLE) patients and may be essential to the disease activity. This study aimed to describe cutaneous manifestations spectrum and determine the association of cutaneous lesions with the disease activity and systemic involvement among SLE patients in Malang, Indonesia.

**Methods:**

A cross-sectional study was conducted using 54 SLE patients from rheumatology outpatient clinic at Saiful Anwar General Hospital Malang, Indonesia. Cutaneous features were classified according to Gilliam and Sontheimer classification of cutaneous lupus. Disease activity and clinical manifestations were documented according to Mexican-SLE disease activity index (Mex-SLEDAI).

**Results:**

Among 54 subjects, 50% of the patients had cutaneous manifestations. Subacute cutaneous lupus erythematosus (SCLE) was observed in 11.1% of patients, and malar rash in 20.4%. Subjects with cutaneous lesions had significantly higher Mex-SLEDAI scores, especially those who had SCLE (p<0.001), malar rash (p=0.002), alopecia (p=0.002), and photosensitivity (p=0.032). Six patients (11.1%) had skin infections with higher disease activity (9[8–11]vs.2[0–4];p<0.001). SCLE was significantly associated with malar rash (OR 11.7[1.8–76.5]), vasculitis (OR 43.0[4.1–445.6]), and fatigue (OR 15.0[2.1–108.8]). Malar rash was associated with photosensitivity (OR 8.4[1.6–44.0]), while oral or nasal ulcer was associated with fatigue (OR 8.6 [1.4–54.6]). Vasculitis (OR 5.9[1.0–35.1]) and nephritis (OR 11.7 [1.8–76.5]) were associated with the presence of skin infection.

**Conclusion:**

SCLE and malar rash are the most common cutaneous lesions among subjects. Subjects with cutaneous lesions have relatively higher disease activity. Several skin lesions are also associated with SLE patients’ systemic manifestations.

## INTRODUCTION

Systemic Lupus Erythematosus (SLE) is a complex autoimmune disorder affecting multiple organs. The clinical manifestations of SLE may range from mild localised skin disease to a life-threatening illness, such as lupus nephritis or neuropsychiatric SLE.^1,2^ The complex interaction among genetic, environmental, and hormonal factors is key to clinical heterogeneity in SLE manifestations. The prevalence and incidence of SLE have been increasing in recent years. Reported Global SLE prevalence was estimated to be 43.7 (15.87 to 108.92) per 100.000 people and affected 3.41 million people respectively.^3^ SLE is more common in women than men, reaching from 2:1 up to 15:1 in the ratio.^4,5^

SLE is often misdiagnosed as another disease since the clinical manifestations of SLE are similar to other conditions.^6,7^ Skin is the most commonly affected organ in a patient with SLE. About 85% of patients with SLE will show cutaneous manifestations at some point during their disease course.^8^ Cutaneous manifestation has been an essential feature of SLE since it was included in the classification criteria of this disease by the American College of Rheumatology (ACR) in 1997 comprises malar rash, discoid rash, oral ulcers, and photosensitivity.^9^

Cutaneous Lupus Erythematosus (CLE) is a wide-spectrum disease with variable evolution. There are still no universal criteria for various subtypes of skin manifestation in SLE.^10^ Skin findings are histopathologically and clinically classified into specific and non-specific, according to Gillam.^11^ LE-specific lesions showed the characteristic of skin manifestation of SLE. LE-specific lesions consist of acute cutaneous LE (ACLE), subacute cutaneous lupus erythematosus (SCLE), and chronic cutaneous lupus erythematosus (CCLE). LE non-specific cutaneous (NSC) included skin manifestations frequently associated with SLE but not specific to the disease, such as cutaneous vasculitis, photosensitivity, urticaria, Raynaud’s phenomenon, livedo reticularis, thrombophlebitis, sclerodactyly, hyperpigmentation, calcinosis cutis, or rheumatoid knuckles. It is a common finding in SLE patients and only occurs during the active phase of the disease.^10–12^

The CLE spectrum is reported to differ between populations and races.^13^ Unfortunately, only a few published reports show the spectrum of CLE in SLE patients from Indonesia. Not only being frequently misdiagnosed, but SLE patients with cutaneous manifestations are also often ignored or under-treated. In contrast, cutaneous lesions are an important marker of SLE disease activity and are associated with internal organ involvement.^14,15^ Therefore, we specifically investigated the prevalence, spectrum, and association of CLE and LE-NSC features with the disease activity and systemic manifestations in SLE patients in an urban city in Indonesia.

## PATIENTS AND METHOD

This cross-sectional study involved fifty-four SLE patients from the Rheumatology outpatient clinic. All patients should fulfill the classification of SLE based on the 2019 EULAR/ACR criteria.^16^ All patients were females aged ≥18 years old, while those who were pregnant or breastfeeding were excluded from the study. Patients were collected using the consecutive sampling method that came to the Rheumatology outpatient clinic in Saiful Anwar General Hospital from June to August 2022. Demographic data, clinical, and laboratory features were obtained from the medical records. The Ethical committee of Saiful Anwar General Hospital Malang approved this study (ethical approval number: 400/204/K.3/102.8/2022, approved at June 2^nd^, 2022), and all subjects signed the written informed consent before the examination.

The rheumatologists or internists performed the diagnosis of SLE and clinical examinations, while the dermatologists observed the cutaneous features. The Gilliam and Sontheimer classification was used to categorise the cutaneous features. The cutaneous features were classified into specific and non-specific cutaneous lesions.^11^ The other cutaneous findings from the patients were also noted. Disease activity was measured using the Mexican-Systemic Disease Activity (Mex-SLEDAI) score. Routine laboratory tests, such as complete blood counts and urinalysis were performed from all subjects. Lupus nephritis was defined according to the clinical criteria (heme-granular or erythrocyte urinary casts, or hematuria, or proteinuria >0.5 gr/24 hours, or pyuria) and confirmed by the renal biopsies showed the characteristics of lupus nephritis. Vasculitis was defined as the presence of ulceration, or gangrene, or tender finger nodules, or periungual infarction, or splinter hemorrhage that was proven by the biopsy or angiogram that showed the signs for vasculitis.^17^ The clinical manifestations that the rheumatologists observed were also documented from the component of the Mex-SLEDAI score.

### Statistical Analysis

Continuous data were presented as mean ± standard deviation (SD), while the categorical data were presented as frequencies and percentages. The median and interquartile range (IQR) was used to explain the continuous data that did not normally distributed. Independent t-test or Mann-Whitney test was performed to compare two continuous variables. On the other hand, Chi-Square or Fisher Exact’s test was used to compare the categorical variables. The association between the variables and the presence of LE cutaneous specific lesions and NSC lesions was done by the logistic regression model. Data were presented as the odd ratio (OR) with 95% confidence interval (CI). A *p*-value <0.05 was considered to be statistically significant. All the statistical analysis was done by the SPSS version 25 for Windows.

## RESULTS

### Subject characteristics

Fifty-four patients were included in this study. Table 1 shows the characteristics of the subjects of this study. Twenty-seven documented patients had cutaneous manifestations according to the Gilliam and Sontheimer classification, while the other twenty-seven had no cutaneous abnormalities. Subjects were divided into two groups, the ones who had cutaneous manifestations and those who did not have any cutaneous manifestations. Both of the groups had similar age distributions. However, patients who were with cutaneous manifestations had more recent disease onset compared to the ones who were without the cutaneous manifestations [24.0 (10.0 – 48.0) vs. 60.0 (24.0 – 84.0) months, p=0.003]. Patients were monitored for clinical manifestations according to the Mex-SLEDAI score. The frequencies of the clinical manifestations from both groups were relatively similar. However, patients with cutaneous manifestations had higher frequencies of fatigue and higher Mex-SLEDAI score, as shown in **Table 1**. The frequency of patients who received methylprednisolone was also higher in patients with cutaneous manifestations. In addition, a higher daily dose of methylprednisolone was found to be significantly higher in patients with cutaneous manifestations (0 [0 – 4] mg/day vs. 4 [0 – 8] mg/day, p=0.038). On the other hand, the frequencies of other medications were relatively similar in both groups.

**Table 1.** Characteristics of the subjects.

**Characteristics**	**Without Cutaneous Manifestations (n = 27)**	**With Cutaneous Manifestations (n = 27)**	**p**
Age (years)	31.2 ± 10.1	32.3 ± 12.1	0.705
Onset of disease (months)	60.0 (24.0 – 84.0)	24.0 (10.0 – 48.0)	0.003*
Clinical manifestations [n (%)]			
- Nephritis	5 (18.5)	6 (22.2)	0.735
- Vasculitis	4 (14.8)	6 (22.2)	0.484
- Arthritis	4 (14.8)	3 (11.1)	0.685
- Thrombocytopenia	0 (0)	2 (7.4)	0.150
- Lymphopenia	2 (7.4)	3 (11.1)	0.639
- Fatigue	0 (0)	6 (22.2)	0.009*
Mex-SLEDAI score	1 (0 – 3)	4 (2 – 8)	<0.001*
Status of the disease [n (%)]			
- Remission	14 (51.8)	0 (0)	<0.001*
- Active disease	13 (48.1)	27 (100)	
Medications [n (%)]			
- Methylprednisolone	8 (29.6)	16 (59.3)	0.028*
- Hydroxychloroquine	24 (88.9)	22 (81.5)	0.444
- Azathioprine	7 (25.9)	5 (18.5)	0.513
- Mycophenolate Mofetil	9 (33.3)	8 (29.6)	0.770
- Cyclosporine	2 (7.4)	1 (3.7)	0.552
- Methotrexate	1 (3.7)	0 (0)	0.313
Daily dose of methylprednisolone (mg/day)	0 (0 – 4)	4 (0 – 8)	0.038*

Mex-SLEDAI: Mexican-Systemic Lupus Erythematosus disease activity index;

* statistically significant for comparison between groups with p<0.05.

### Distribution of skin manifestations and association with disease activity

The distribution of the cutaneous abnormalities or skin manifestations among subjects with SLE is shown in **Table 2**. The type of skin manifestations was classified into two categories: specific skin lesions and non-specific skin lesions according to the Gilliam and Sontheimer classification. Subacute cutaneous lupus erythematosus (SCLE) was identified in 11.1% of the patients, while 20.4% had localized skin manifestations (malar rash). Alopecia was the most prevalent non-specific skin lesion in 46.3% of patients with SLE. We also noted that the patients had other cutaneous manifestations other than specific and non-specific skin lesions. Other skin lesions that were found in our SLE patients were post-inflammatory hyperpigmentation (29.6%), striae (33.3%), and skin infections (11.1%). The types of skin infections that were found were tinea (3 patients), scabies (2 patients), and verrucae (1 patient).

**Table 2.** Distribution of skin manifestations and the association with disease activity.

**Type of Skin Manifestations**	**Frequencies [n (%)]**	**Mex-SLEDAI Score**		**p**

		**Absent**	**Present**	
*Specific skin lesion*				
- Subacute cutaneous lupus erythematosus	6 (11.1)	2 (0 – 4)	9 (7 – 11)	<0.001*
- Localised (malar rash)	11 (20.4)	2 (0 – 4)	8 (4 – 9)	0.002*
*Non-specific skin lesion*				
- Alopecia	25 (46.3)	2 (0 – 4)	4 (2 – 8)	0.002*
- Oral or nasal ulcer	8 (14.8)	2 (0 – 6)	4 (3 – 6)	0.205
- Raynaud’s phenomenon	6 (11.1)	2 (1 – 6)	6 (4 – 11)	0.086
- Photosensitivity	24 (44.4)	2 (0 – 4)	4 (2 – 8)	0.032*
- Urticaria	5 (9.3)	2 (1 – 6)	2 (0 – 9)	0.977
*Other skin lesion*				
- Post-Inflammatory Hyperpigmentation (PIH)	16 (29.6)	2 (0 – 7)	2 (2 – 4)	0.423
- Striae	18 (33.3)	3 (2 – 6)	2 (0 – 6)	0.317
- Infection	6 (11.1)	2 (0 – 4)	9 (8 – 11)	<0.001*

Mex-SLEDAI: Mexican-Systemic Lupus Erythematosus disease activity index; PIH=post-inflammatory hyperpigmentation;

* statistically significant for comparison between groups with p<0.05.

A comparison of the disease activity was made according to the presence of the subjects’ skin manifestations, as seen in **Table 2**. Patients with SCLE (Mex-SLEDAI score 9 [7 – 11] vs. 2 [0 – 4], p<0.001) and malar rash (Mex-SLEDAI score 8 [4 – 9] vs. 2 [0 – 4], p=0.002) had significantly higher of Mex-SLEDAI score. As for the non-specific skin lesions, subjects who had alopecia (Mex-SLEDAI score 4 [2 – 8] vs. 2 [0 – 4], p=0.002) and photosensitivity (Mex-SLEDAI score 4 [2 – 8] vs. 2 [0 – 4], p=0.032) showed a significantly higher of Mex-SLEDAI score compared to the ones who did not present these manifestations. Subjects who developed skin infections also had markedly higher disease activity (Mex-SLEDAI score 9 [8 – 11] vs. 2 [0 – 4], p<0.001).

### Association of cutaneous lesions with other clinical manifestations in SLE patients

The association between the cutaneous lesions with other clinical manifestations among the subjects is shown in **Table 3**. Only variables with significant association were shown in **Table 3**. Malar rash (OR 11.7 95% CI [1.8 – 76.5], p=0.003), vasculitis (OR 43.0 95% CI [4.1 – 445.6], p<0.001), and fatigue (OR 15.0 95% CI [2.1 – 108.8], p=0.001) were associated with the presence of SCLE. On the other hand, photosensitivity was significantly related to malar rash (OR 8.4 95% CI [1.6 – 44.0], p=0.005). Oral or nasal ulcer was associated with fatigue (OR 8.6 95% CI [1.4 – 54.6], p=0.010). Skin infection was also a serious problem that might occur in SLE patients. Therefore, we found that the presence of vasculitis (OR 5.9 95% CI [1.0 – 35.1], p=0.035) and nephritis (OR 11.7 95% CI [1.8 – 76.5], p=0.003) was strongly associated with the skin infection.

**Table 3.** Association of cutaneous lupus with other clinical manifestations among subjects.

**Variables**	**Present (n = 6)**	**Absent (n = 48)**	**OR (95% CI)**	**p**
Subacute Cutaneous Lupus Erythematosus				
- Malar rash	4 (66.7)	7 (14.6)	11.7 (1.8 – 76.5)	0.003*
- Vasculitis	5 (83.3)	5 (10.4)	43.0 (4.1 – 445.6)	<0.001*
- Fatigue	3 (50)	3 (6.3)	15.0 (2.1 – 108.8)	0.001*

Localised (Malar Rash)	*Present (n = 11)*	*Absent (n = 43)*		
- Photosensitivity	9 (81.8)	15 (31.3)	8.4 (1.6 – 44.0)	0.005*

Oral or Nasal Ulcer	*Present (n = 6)*	*Absent (n = 48)*		
- Fatigue	3 (50)	3 (6.3)	8.6 (1.4 – 54.6)	0.010*

Skin Infection	*Present (n = 6)*	*Absent (n = 48)*		
- Vasculitis	3 (50.0)	7 (14.6)	5.9 (1.0 – 35.1)	0.035*
- Nephritis	4 (66.7)	7 (14.6)	11.7 (1.8 – 76.5)	0.003*

* statistically significant for the association of other clinical manifestations with cutaneous lupus manifestations.

## DISCUSSION

SLE is a multiorgan autoimmune disease. The skin is one of the main target organs of this disease.^8^ This study found that 50% of patients had skin symptoms with predominantly SCLE and malar rash. The previous research with Indonesian SLE patients also showed similar results, with the prevalence of mucocutaneous manifestation found in 30–60% of patients.^18,19^ A similar distribution was also found in the previous study that showed the malar rash and SCLE was the predominant lesion in Indonesian SLE patients.^2^ Our findings showed that the cutaneous manifestations were more frequent in subjects with more recent disease onset of SLE. Similar results also demonstrated in the Thailand population that the CLE was more frequent in adult-onset SLE patients than in late-onset SLE patients.^20^ A systematic review and meta-analysis also showed that cutaneous manifestations were less common in late-onset SLE patients compared to early-onset patients.^21^ Several factors, such as immunogenetics, immunosenescence, medications, or environment, still need to be investigated to understand this phenomenon clearly.

Our findings also demonstrated higher disease activity in subjects with cutaneous manifestations, specifically subjects with SCLE, malar rash, alopecia, and photosensitivity. In a previous study, higher disease activity was associated with CLE and non-specific cutaneous lesions.^20^ Cutaneous lesions also had been demonstrated as an excellent diagnostic value in SLE. Malar rash might become a marker of more disease activity of SLE that was reported in the previous study.^22^ Zecević et al. demonstrated that patients with lupus-nonspecific lesions had significantly more active SLE and required more intensive therapy and disease monitoring.^23^ Although the mechanisms that explained the association between the CLE and SLE disease activity were still not clearly understood, about 10% of patients with CLE-only might develop into SLE with systemic manifestations. In addition, CLE patients with non-specific cutaneous lesions were associated with high antinuclear antibody (ANA) titre, renal, hematologic, joint involvement, and greater SLEDAI score.^24^

The presence of CLE was associated with other systemic manifestations. ACLE commonly occurs in patients with lupus nephritis.^25^ Koch and Tikly demonstrated that ACLE was strongly associated with renal disease, and the discoid rash was associated with arthritis and Raynaud’s phenomenon.^26^ Fatigue was a vital symptom commonly affecting SLE patients and negatively affected the patient’s quality of life. Similar to our study, Tarazi et al. demonstrated that SLE patients with skin disease experienced more fatigue than controls.^27^

Skin infection was also one of the significant problems that SLE patients experienced. A prior study showed that almost 22.5% of SLE patients developed a skin infection.^28^ Most studies showed that the prescribed treatment caused the infection.^29–31^ However, we did not find any association between the medication used in this study and skin infection. Our findings demonstrated that higher disease activity was shown in subjects who had an infection on their skin. In addition, we found that skin infection was associated with renal disease and vasculitis. Mok et al. demonstrated that the incidence of herpes zoster was common in lupus nephritis patients but still unpredictable to the disease activity.^32^ Immune dysregulation due to high disease activity still significantly increases the susceptibility to infection among SLE patients.^33^ Despite this positive finding, it is still difficult to conclude the association between the disease activity or systemic manifestation with skin infection from this study. Overall, this study contributes to the existing knowledge by providing insights into the relationship between the spectrum of CLE, disease activity, and systemic manifestations in patients with SLE. The findings of this study might have clinical implications for the management and treatment of SLE patients. By recognising the specific subtypes of CLE that were associated with higher disease activity and systemic involvement, healthcare professionals could tailor their approaches to better monitor and address the needs of SLE patients, potentially leading to improved outcomes. Another aspect addressed in the study was the association between the different forms of CLE and systemic manifestations in SLE patients. By examining the relationship between cutaneous involvement and systemic features, the study aimed to elucidate the potential impact of CLE on the overall disease burden and prognosis of SLE patients.

However, our study also possesses several limitations. First, this is a single-centre study with a homogeneous population, mainly from Java Island in Indonesia. Therefore, these findings still cannot describe Indonesia’s whole population. Second, the number of subjects in this study is relatively small. Thus, we need to expand the subject population to a larger number to obtain a better causal-effect relationship between the cutaneous manifestations and the disease activity. Lastly, there was also lack of serological profiles and histopathological data form the subjects that could indeed be considered as a limitation for this study. Including such descriptive information could provide valuable insights into the disease pathology and help establish a clearer understanding of the patients’ conditions. It may also contribute to the overall validity and comprehensiveness of the study’s findings. In conclusion, we described that the spectrum of cutaneous manifestation in our SLE patients was similar to other populations in Indonesia compared to previous studies. In addition, subjects with cutaneous manifestations had a relatively higher disease activity. Several cutaneous findings are also associated with systemic organ manifestations. These findings described the importance of the cutaneous manifestations as the marker of disease activity, and physicians should be more aware of treating patients with cutaneous manifestations to achieve the therapeutic target in SLE.
